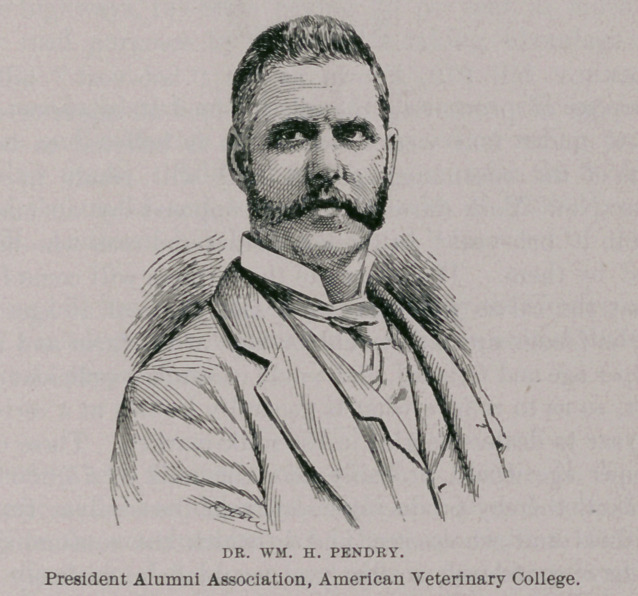# American

**Published:** 1898-05

**Authors:** 


### AMERICAN VETERINARY COLLEGE.

For the first time in many years the American Veterinary College held no formal exercises. The innovation of 1898, of conferring the degrees within the college walls, is likely to advance the college interests more, perhaps, than formal exercises in a public hall. It affords a better opportunity of linking this completed step of the undergraduate with a closer acquaintance and affiliation with the older graduates, and affords an opportunity of ushering in the added alumni amidst those who yearly return to their college parent to renew their devotion to her interests and to learn how better her progress may be advanced along new lines. While the adding of the present year's class was done under many disadvantages, it is to be hoped that next year, in following this departure, it may be made an occasion of rare pleasure and commingling among those who long to see the A. V. C. prosper.

The degree of D.V.S. (doctor of veterinary surgery) was conferred by the President of the Board of Tustees, Prof. Faneuil D. Wiesse, and the delivery of the diplomas to each one of the graduating class by Dean W. J. Coates. The President of the Board also announced the successful winners of the several prizes offered for competition. The following gentlemen received the degree of the college: Charles Steward Atchison, Brooklyn, N. Y.; Walter Gideon Biehl, Loyalsock, Pa.; William Franklin Braisted, Port Richmond, N. Y.; Peter Thomas Bergen, Fordham, N. Y.; John Mason Broadwell, Morristown, N. J.; John Francis DeVine, Rhinebeck, N. Y.; Howard Julius Earl, Natick, Mass.; George Percy Ellice, Jersey City, N. J.; R. W. A. English, Jersey City, N. J.; Edward Charles Fox, Baltimore, Md.; John Frederick Fausner, New York, N. Y.; William Henry Hogan, Bayonne, N. J.; William Lawrence Johnson, Brooklyn, N. Y.; Lester R. J. Limbeck, Jersey City, N. J.; James Jerome Molony, Brooklyn, N. Y.; Charles Henry Myers, Middletown, Conn.; Andrew Raphael Morris, New York, N. Y.; Joseph Franklin Price, Cogan Station, Pa.; Adolph John Pistor, Jr., Newark, N. J.; Wilbur John Southey, Bridge port, Conn.; Edward Fairchild Sanford, Oxford, Conn.; Robert Allen Stimpson, Port Henry, N. Y.; Charles Elmer Ellsworth Tomlinson, Williamsport, Pa.; Roger Irving Twombly, Alton, N. H.; James Washington Walker, Brooklyn, N. Y.; George Weisbrod, Brooklyn, N. Y.

John Francis DeVine, having passed the best general examination, received the Trustees' gold medal. Adolph John Pistor, Jr., having passed the second-best general examination, and Edward Fairchild Sanford the next-best examination, also received prizes. John Francis DeVine received the faculty's gold medal for best practical examination before a committee of three practising veterinarians of New York and Brooklyn. The prize of Dr. Liautard, for the best anatomical specimen prepared by a member of the graduating class, was awarded to Edward Charles Fox. W. Fretz, of the second-year class, having obtained the greatest proficiency, was awarded the free scholarship for the year 1898–99, and W. A. Young, being the most proficient in the first-year class, secured Dr. Liautard's medal for greatest proficiency in junior anatomy.

After conferring the degrees, at 4.30 P.M. the alumni association convened (March 31, 1898). President Howard called the meeting to order and the names of the members of the various classes represented were recorded. Secretary Faust read the minutes of the meeting of 1897, and the special and regular meetings of the Executive Committee announcing the provision of the alumni prizes, the annual banquet, and the selection of W. Horace Hoskins, of the class of '81, as toastmaster. President Howard received the graduates of '98 as members of the alumni association, and welcomed them with the hope that their interests in association affairs and college work would promote her interests, and that they would lead in the movement in 1900, our twenty-fifth anniversary, to make it an important epoch in the college's history. He urged the importance of maturing the plans at once for this celebration, and asked for the freest discussion of such plans as would fittingly celebrate this event. Drs. W. Herbert Lowe, William H. Pendry, E. B. Ackerman, H. D. Hanson, W. Horace Hoskins, and others, discussed the subject, and suggested many features and propositions, all of which were, by vote, referred to the incoming officers and Executive Committee with power to act. Interesting reports of our alumni in Maryland and Pennsylvania were received and much appreciated by all who had the pleasure of hearing them.

The death of A. Stein, M.D., emeritus professor of physiology, was formally announced and feelingly referred to, after which suitable resolutions relative to the great loss were adopted and ordered to be engrossed upon the minute-book.

The deaths of our fellow members, Theodore Birdsall, class of '81; E. Oscar Busener, class of '91, and C. Chaney Tietjens, class of '97, were announced and appropriate resolutions offered and adopted and ordered engrossed upon the minutes.

The election of officers resulted in the selection of Dr. Wm. H. Pendry, of Brooklyn, class of '83, as President, a well-deserved recognition of one who has been faithful and loyal to college, association, and professional interests in season and out of season. For First Vice-President, Dr. Robert W. Ellis, of New York City, class of '89, and for Second Vice-President, Dr. Warren L. Rhoads, of Lansdowne, Pa., class of '95, both active and earnest association workers. The efficient services of Dr. F. R. Hanson, of New York City, class of '95, as Treasurer, resulted in his re-election for this place. Dr. C. E. Clayton, of New York City, class of '93, was selected as Secretary, it being desired to have this office in closer touch with the college in view of the approaching twenty-fifth anniversary. Dr. Sanford, class of '98, was elected Librarian.

DR. WM. H. PENDRY.

President Alumni Association, American Veterinary College.

Alumni Trustee, W. Herbert Lowe, reported the meetings of the Board of Trustees, and referred to expected changes in the Regents' examination that would be of much importance to the welfare of the institution.

The selection of Dr. Robert W. Ellis as lecturer on obstetrics, Dr. C. E. Clayton, as assistant to the chair of surgery, Dr. E. C. Beckett, as lecturer on zoötechny, and Prof. Olof Schwarzkopf, as professor of bacteriology, for the ensuing year, as faculty changes, were announced and received with much approbation and appreciation.

At 7 P.M. many faces were turned toward the Hotel Marlborough, where at 7.30 P.M. the twenty-third annual banquet of the alumni association was spread, and those who were privileged to join in the feast found much pleasure and enjoyment. Some thirteen classes were represented, extending from 77' to '98 inclusive, and many were the happy responses brought out by Toastmaster Hoskins from Dean Coates of '77; ex-President Howard, of '82; Arrowsmith, of '83; J. E. Ryder, of '84; Editor Bell, of '87; W. H. Lowe and Decker ,of '88; H. D. Hanson and Ellis, of '89; Ackerman, of '91; Clayton, of '92; Butler, of '95; Leary, of '96, and members Weisbrod and Walker, of the class of '98, brought forth many rich things from their class-members, in class-history, class-songs, and college experiences, making it a very merry evening and fitting close to our college year of '98.

At a late hour the arrival of Prof. J. B. Stein proved a pleasure of much moment, and his thoughtful words about college work, laboratory needs, and how these grew in value from small beginnings, found an echo of approval and appreciation in the hearts of every one present.

## Figures and Tables

**Figure f1:**